# Preventative therapeutic approaches for hypertrophic cardiomyopathy

**DOI:** 10.1113/JP279410

**Published:** 2020-09-07

**Authors:** Tanya Solomon, Aleksandra Filipovska, Livia Hool, Helena Viola

**Affiliations:** ^1^ School of Human Sciences University of Western Australia Crawley Western Australia Australia; ^2^ Harry Perkins Institute of Medical Research QEII Medical Centre Nedlands Western Australia Australia; ^3^ ARC Centre of Excellence in Synthetic Biology QEII Medical Centre Nedlands Western Australia Australia; ^4^ Centre for Medical Research University of Western Australia QEII Medical Centre Nedlands Western Australia Australia; ^5^ Telethon Kids Institute Perth Children's Hospital Nedlands Western Australia Australia; ^6^ School of Molecular Sciences University of Western Australia Crawley Western Australia Australia; ^7^ Victor Chang Cardiac Research Institute Sydney New South Wales Australia

**Keywords:** hypertrophic cardiomyopathy, metabolism, mitochondria, therapy

## Abstract

Sarcomeric gene mutations are associated with the development of hypertrophic cardiomyopathy (HCM). Current drug therapeutics for HCM patients are effective in relieving symptoms, but do not prevent or reverse disease progression. Moreover, due to heterogeneity in the clinical manifestations of the disease, patients experience variable outcomes in response to therapeutics. Mechanistically, alterations in calcium handling, sarcomeric disorganization, energy metabolism and contractility participate in HCM disease progression. While some similarities exist, each mutation appears to lead to mutation‐specific pathophysiology. Furthermore, these alterations may precede or proceed development of the pathology. This review assesses the efficacy of HCM therapeutics from studies performed in animal models of HCM and human clinical trials. Evidence suggests that a preventative rather than corrective therapeutic approach may be more efficacious in the treatment of HCM. In addition, a clear understanding of mutation‐specific mechanisms may assist in informing the most effective therapeutic mode of action.

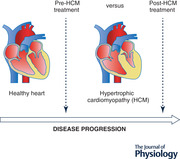

## Graphical abstract

Sarcomeric gene mutations are associated with the development of hypertrophic cardiomyopathy (HCM). Mechanistically, alterations in calcium handling, sarcomeric disorganization, energy metabolism and contractility participate in HCM disease progression. These alterations may precede or proceed development of the pathology. This review assesses the efficacy of preventative *versus* corrective HCM therapeutics from studies performed in animal models of HCM and human clinical trials.

## Introduction

Hypertrophic cardiomyopathy (HCM) is an autosomal dominant cardiovascular disease that affects 1:200 of the general population (Semsarian *et al*. 2015). It is well‐documented that the clinical characteristics of HCM consist of left ventricular wall thickening in the absence of increased haemodynamic workload, diastolic dysfunction and in other cases left ventricular outflow tract (LVOT) obstruction, mitral valve abnormalities and left atrial enlargement (Maron *et al*. 2006; Marian & Braunwald, 2017). At the cellular level, HCM is characterized by cardiac myocyte remodelling, disorganization of sarcomeric proteins, interstitial fibrosis and altered energy metabolism (Watkins *et al*. 2011). The literature to date suggests that HCM occurs primarily due to genetic mutations in sarcomeric proteins, which demonstrate variable penetrance and heterogeneous phenotypic expression in patients (Marian & Braunwald, 2017).

**Figure 1:**
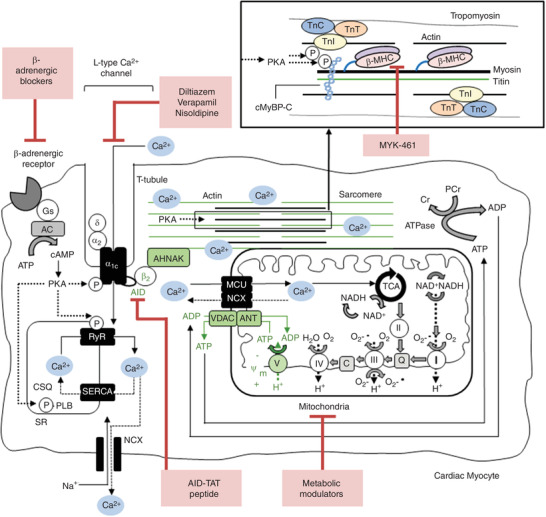
Current therapeutic targets in the treatment of hypertrophic cardiomyopathy The cardiac L‐type calcium channel is comprised of α_1C_, α_2_δ and β_2_ subunits. Upon β‐adrenergic receptor stimulation, calcium (Ca^2+^) influx through the pore‐forming α_1C_ subunit initiates ‘Ca^2+^‐induced‐Ca^2+^‐release’ from sarcoplasmic reticulum (SR) stores via the ryanodine receptor (RyR). ATP production occurs through Ca^2+^‐dependent mitochondrial oxidative phosphorylation, a process involving Ca^2+^ uptake by the mitochondrial Ca^2+^ uniporter (MCU), subsequent activation of the tricarboxylic acid (TCA) cycle and movement of electrons down complexes I–V of the electron transport chain. The β_2_ subunit of the L‐type calcium channel is anchored to F‐actin via subsarcolemmal stabilizing protein AHNAK. Mitochondria also associate with F‐actin via mitochondrial docking proteins. Mechanism for calcium‐independent regulation of mitochondrial membrane potential (Ψ_m_) by the L‐type calcium channel is shown in green. Concurrently, Ca^2+^ binds to thin filaments, which, powered by ATP, results in contraction. During the course of contraction, ATP is converted to ADP via ATPase, and back to ATP via the conversion of phosphocreatine (PCr) to creatine (Cr). Actin–myosin interaction is regulated by the cardiac troponin (cTn) complex, which is anchored to tropomyosin by cardiac troponin T (cTnT). When Ca^2+^ binds to cardiac troponin C (TnC), cTnI undergoes a conformational change that allows actin–myosin interaction, and therefore contraction. During relaxation, cTnI inhibits actin–myosin interaction. Cardiac myosin binding protein‐C (cMyBP‐C) binds to actin, myosin and titin and plays a role in regulating actin–myosin cross‐bridge cycling and contractility. Hypertrophic cardiomyopathy (HCM) is caused by mutations in sarcomeric proteins. Existing therapeutic targets for treatment of HCM include β‐adrenergic receptor (blockers), L‐type calcium channel blockers (diltiazem, verapamil and nisoldipine), and β‐myosin heavy chain (β‐MHC)/actin binding (Mavacamten, MYK‐461). AC, adenylyl cyclase; AID, alpha‐interaction domain; cAMP, cyclic adenosine monophosphate; CSQ, calsequestrin; Gs, G‐stimulatory protein; NCX, sodium/calcium exchanger; P, phosphorylation; PKA, protein kinase A; PLN, phospholamban; SERCA, sarcoplasmic reticulum Ca^2+^‐ATPase. Adapted from Viola & Hool (2019).

In cardiac muscle, calcium influx through the L‐type calcium channel (I_Ca‐L_) in response to depolarization of the plasma membrane initiates contraction which leads to complex interactions between sarcomeric proteins and sarcoplasmic reticulum Ca^2+^ release. Genetic studies have identified over 1500 different mutations in genes of sarcomere proteins that have been associated with the development of HCM (Marian & Braunwald, 2017). The most common mutations appear to be cardiac myosin binding protein‐C (*MYBPC3*), β‐myosin heavy chain (*MYH7*), troponin I (*TNNI3*), troponin T (*TNNT2*) and α‐tropomyosin (*TPM1*) (Watkins *et al*. 1995; Seidman & Seidman, 2001; Sabater‐Molina *et al*. 2018). Regarding function, β‐myosin heavy chain (β‐MHC) is a sarcomeric protein that consists of a myosin carboxyl terminal rod domain, and an amino terminal globular head domain that interacts with actin filaments during muscle contraction (Fig. 1) (Rayment *et al*. 1993; Sata *et al*. 1997). Actin–myosin interactions that occur during excitation–contraction coupling, are regulated by the cardiac troponin (cTn) complex (Chandra *et al*. 2007). Cardiac troponin is composed of three regulatory subunits: cardiac troponin I (cTnI), cardiac troponin T (cTnT) and cardiac troponin C (cTnC). Cardiac troponin I regulates cardiac contraction and relaxation in response to alterations in intracellular calcium (Ca^2+^), while cTnT anchors the entire cTn complex to tropomyosin (Cheng & Regnier, 2016). During relaxation, cTnI inhibits the actin–myosin interaction, but when Ca^2+^ binds to the cTnI Ca^2+^ binding site (cTnC), cTnI undergoes a conformational change that allows the actin–myosin interaction and as a result, contraction (Cheng & Regnier, 2016). Cardiac myosin binding protein‐C (cMyBP‐C) is a thick filament associated protein that is believed to have structural importance by binding to actin, myosin and titin, as well as functional importance, through regulation of cross‐bridge cycling and cardiac muscle contractility (Freiburg & Gautel, 1996; Sequeira *et al*. 2014).

Evidence suggests that HCM‐causing sarcomeric gene mutations are associated with disorganization of sarcomere proteins, alterations in Ca^2+^ handling, myofilament Ca^2+^ sensitivity and mitochondrial metabolic function (for a review, see Viola & Hool, 2019). Indeed, patients expressing sarcomeric gene mutations are found to have decreased myocardial energy efficiency, which is thought to play an important role in the molecular pathway of the disease. However, although some similarities exist, each mutation appears to result in mutation‐specific pathophysiology (Ferrantini *et al*. 2017; Viola & Hool, 2019). Additionally, these alterations may precede or proceed development of HCM pathology. This may contribute to the observed phenotypic variability in sarcomeric‐related HCM, and as a result, provide an additional challenge to the design of effective drug therapy. Recent findings indicate that the cardiac L‐type calcium channel (I_Ca‐L_) and mitochondria may play a collaborative role in the development of HCM (Viola & Hool, 2019). Interestingly, this appears to occur before the development of the pathology. In the present article, we assess the current knowledge regarding hypertrophic cardiomyopathy therapeutics in order to develop an understanding of the efficacy of preventative compared to corrective approaches (Abstract Figure).

## Role of the L‐type calcium channel in cardiac function

Calcium entry into cardiac myocytes through the I_Ca‐L_ is critical for maintaining cardiac excitation and contraction (Bodi *et al*. 2005). The I_Ca‐L_ is a heterotetrameric structure consisting of the pore‐forming α_1C_ and the accessory β_2_ and α_2δ_ subunits (Fig. 1). The α_1C_ subunit is a transmembrane structure consisting of four homologous motifs that regulate ion conductance and voltage sensing and contains binding sites for channel‐modifying second messengers, toxins and drugs (Bodi *et al*. 2005). The β_2_ subunit of the I_Ca‐L_ is entirely intracellular and assists with trafficking and insertion of the α_1C_ subunit in the cell membrane (Buraei & Yang, 2013). The β_2_ subunit is bound to the cytoplasmic I–II linker of the α_1C_ subunit of the channel called the alpha‐interaction domain (AID) and undergoes conformational movement during channel activation and inactivation (Bodi *et al*. 2005).

The I_Ca‐L_ regulates mitochondrial function via both Ca^2+^‐dependent and Ca^2+^‐independent mechanisms (Fig. 1). *In vitro* studies using intact quiescent cardiac myocytes, demonstrate that activation of the I_Ca‐L_ by voltage‐clamp of the plasma membrane, or the I_Ca‐L_ agonist BayK(−), leads to increased intracellular Ca^2+^, increased mitochondrial Ca^2+^ uptake and superoxide production, and increased mitochondrial metabolic activity (Viola *et al*. 2009). While each of these responses is Ca^2+^‐dependent, there is also evidence that activation of the I_Ca‐L_ results in increased mitochondrial membrane potential (Ψ_m_) that occurs in a Ca^2+^‐independent manner (Viola *et al*. 2009, 2016*a*). This response may be in part dependent on a structural–functional interaction between the I_Ca‐L_ and mitochondria that is transmitted via sarcomeric proteins.

In cardiac myocytes, microtubules (tubulin), microfilaments (actin) and intermediate filaments, extend from the plasma membrane to traverse cellular organelles including the t‐tubules, sarcoplasmic reticulum and mitochondria (Tokuyasu *et al*. 1983). The β_2_ subunit of the I_Ca‐L_ is anchored to F‐actin networks (Fig. 1) (Rueckschloss & Isenberg, 2001; Hohaus *et al*. 2002). Changes in actin filament organization are sufficient to alter channel kinetics (Haase *et al*. 1999; Hohaus *et al*. 2002; Leach *et al*. 2005). Mitochondria also associate with sarcomeric proteins via mitochondrial docking proteins (Rappaport *et al*. 1998). We have identified that alterations in cardiac I_Ca‐L_ activity can regulate Ψ_m_ via sarcomeric proteins in a Ca^2+^‐independent manner (Viola *et al*. 2009). Preventing movement of the β_2_ subunit with application of a peptide derived specifically against the AID region of the I_Ca‐L_, attenuates increases in Ψ_m_ caused by application of BayK(−) (Viola *et al*. 2009). Additionally, exposure of cardiac myocytes to F‐actin depolymerizing agent latrunculin A also attenuates the response (Viola *et al*. 2014). These findings suggest that the I_Ca‐L_ may influence cardiac mitochondrial function through a structural–functional communication. In support of this concept, the actin cytoskeleton plays an important role in mediating regulation of mitochondrial function by neuronal I_Ca‐L_ (Johnson & Byerly, 1993; de Oliveira *et al*. 2019; Hotka *et al*. 2020). In neurons of the locus coeruleus, application of the mitochondrial protonophore carbonyl cyanide *m*‐chlorophenylhydrazone has been demonstrated to induce a hyperpolarizing response that can be inhibited by application of either I_Ca‐L_ blockers (nifedipine or nicardipine) or the actin depolymerizing agent cytochalasin D (de Oliveira *et al*. 2019). These findings suggest that a structural–functional communication between I_Ca‐L_ and mitochondria may also play a role in regulating neuronal function.

## Role of the L‐type calcium channel and mitochondria in hypertrophic cardiomyopathy disease progression

Mutations in *MYBPC3* genes coding for cMyBP‐C are the most abundant, including primarily heterozygous nonsense mutations, insertions or deletions, and splicing point mutations (Carrier *et al*. 2015). Generally, these mutations result in C‐terminally truncated cMyBP‐C that lacks binding sites for sarcomeric proteins myosin and titin (Carrier *et al*. 2015). Studies performed in murine models expressing these mutations reveal that the absence of cMyBP‐C protein is associated with increased actin–myosin cross‐bridge cycling, myocyte disarray and fibrosis (Harris *et al*. 2002; Carrier *et al*. 2004). HCM patients with these mutations present with a mild disease phenotype and late onset of disease (Barefield *et al*. 2014). Since HCM is associated with disorganization of sarcomeric proteins and altered energy metabolism, it may be reasonable to postulate that a communication ‘breakdown’ between the cardiac I_Ca‐L_ and mitochondria may be involved in progression of HCM.

Transgenic mouse models of HCM are a useful tool to gain further insight into HCM pathophysiology. However, a clear understanding of the underlying mechanisms of disease progression, from a pre‐ to post‐hypertrophic state, has been difficult to ascertain from the current literature. This is in part due to the lack of clarity of cohort age (Viola & Hool, 2019). Therefore, the most valuable knowledge on the role of the I_Ca‐L_ and mitochondria in early and late stage HCM has been gained from studies performed in mouse models of the disease resulting from sarcomeric gene mutations.

In humans, a missense mutation in the *TNNI3* gene encoding the cTnI protein (Gly203Ser) is characterized primarily by the development of apical hypertrophy, and in some cases supraventricular and ventricular arrhythmias (Kimura *et al*. 1997). Transgenic mice with a human disease‐causing Gly203Ser mutation (cTnI‐G203S) develop similar characteristic HCM features by 21 weeks of age, including hypertrophy, hypercontractility, cardiac myocyte disorganization and interstitial fibrosis (Tsoutsman *et al*. 2006; Viola *et al*. 2016*a*).

Cardiac myocytes isolated from 25‐ to 30‐week‐old cardiomyopathic cTnI‐G203S mice exhibit significantly faster I_Ca‐L_ inactivation rates compared to wild‐type myocytes (Viola *et al*. 2016*a*). In addition, consistent with the human phenotype, cardiac myocytes exhibit a hypermetabolic state compared to wild‐type myocytes, as evidenced by significantly larger increases in mitochondrial activity and Ψ_m_ in response to exposure of myocytes to BayK(−) (Viola *et al*. 2016*a*). Interestingly, the increase in Ψ_m_ was not due to further increases in mitochondrial Ca^2+^ uptake in myocytes. We proposed that a structural–functional ‘breakdown’ between the cardiac I_Ca‐L_ and mitochondria may be involved in progression of the disease state. Furthermore, the same responses were observed in myocytes isolated from 10‐ to 15‐week‐old pre‐hypertrophic cTnI‐G203S mice (Viola *et al*. 2016*a*), indicating that altered metabolism appears to occur before the onset of clinical manifestations of HCM.

Patients carrying the Arg403Gln missense mutation in the *MYH7* gene progressively develop septal hypertrophy and myocardial dysfunction and have a high incidence of sudden cardiac death (SCD) (Geisterfer‐Lowrance *et al*. 1990; McConnell *et al*. 2001). There are two cardiac isoforms of MHC: α‐MHC and β‐MHC. The predominant isoform in humans is β‐MHC, accounting for >90% of ventricular myosin (Gupta, 2007). In neonatal mice the predominant isoform is β‐MHC, but expression of β‐MHC is silenced after birth and the predominant isoform transcribed shifts to α‐MHC in adult mice (Gupta, 2007). Heterozygous mice expressing the human Arg403Gln β‐MHC mutation (αMHC^403/+^) gradually develop hypertrophy, myocyte disarray and increased myocardial fibrosis, mimicking the human disease (Geisterfer‐Lowrance *et al*. 1996; Fatkin *et al*. 2000). Myocyte disarray appears to be an early cellular response, while histopathological features such as the development of hypertrophy and fibrosis occur after haemodynamic abnormalities (Geisterfer‐Lowrance *et al*. 1996).

Similar to findings observed in myocytes isolated from cTnI‐G203S mice, myocytes isolated from 30‐ to 50‐week‐old αMHC^403/+^ mice with established hypertrophy and fibrosis exhibit faster I_Ca‐L_ inactivation rates, and a hypermetabolic state compared to wild‐type myocytes (Viola *et al*. 2016*b*). Additionally, myocytes isolated from 10‐ to 15‐week‐old pre‐hypertrophic αMHC^403/+^ exhibit alterations in I_Ca‐L_ inactivation rates, mitochondrial activity and Ψ_m_ which were comparable to those observed in post‐hypertrophic αMHC^403/+^ mice (Viola *et al*. 2016*b*). Consistent with this, *ex vivo* studies assessing pre‐cardiomyopathic 20‐ to 24‐week‐old Arg403Gln mice demonstrate lower cardiac phosphocreatine (PCr) to ATP (PCr/ATP) ratio, indicative of inefficient metabolic energetics (Spindler *et al*. 1998). Overall, data from both cTnI‐G203S and αMHC^403/+^ mice suggest that alterations in I_Ca‐L_ kinetics, and a resulting hypermetabolic state, manifest before the development of the cardiomyopathy. Therefore, targeting the I_Ca‐L_ as a means of normalizing mitochondrial metabolic activity may be an attractive therapeutic approach for the treatment of HCM.

## Evaluation of current hypertrophic cardiomyopathy therapeutics

Clinical studies examining phenotypic heterogeneity in HCM have established that the disease ranges from asymptomatic or mildly symptomatic to severe manifestations (Marian & Braunwald, 2017). The presentation of HCM is age‐dependent and while most patients have a normal life‐expectancy with manageable symptoms, some are at increased risk of heart failure (HF) and SCD (Marian & Braunwald, 2017). Clinical features in patients with HCM, in addition to left ventricular hypertrophy, include altered ejection fraction, atrial fibrillation, ventricular arrhythmias and mitral regurgitation (Marian & Braunwald, 2017). One‐third of patients present with LVOT obstruction at rest, and it can be induced in another third by increased cardiac workload (e.g. exercise) (Maron *et al*. 2006). To date, common therapeutics for patients with HCM focus on symptom management and the prevention of thrombotic events and SCD. These treatment strategies consist primarily of pharmacological therapies, and in more severe cases, surgical interventions including septal reduction and implantable cardioverter‐defibrillators (Spoladore *et al*. 2012). Septal reduction methods such as septal myectomy or septal ablation, can improve function by relieving LVOT obstruction (Spoladore *et al*. 2012). However, these surgical procedures are invasive, target only symptomatic features, are not widely accessible and carry risk to the patients. Additionally, implantable cardioverter‐defibrillators are only used in high‐risk patients, or those with very severe symptoms for the prevention of SCD.

Current pharmacological treatments in patients with HCM mainly aim to reduce LVOT obstruction and increase filling capacity (Ammirati *et al*. 2016). The most widely used therapeutics for HCM include β‐adrenergic receptor blockers and Ca^2+^ channel blockers. Despite some management of symptoms with these drugs, their use can have pleiotropic effects and inconsistent therapeutic responses in patients (Ammirati *et al*. 2016). Given mutation‐specific variations in disease progression (Ferrantini *et al*. 2017; Viola & Hool, 2019), we examined the current knowledge gained from studies performed in both animal models of HCM and clinical trials to develop an understanding of the efficacy of preventative *versus* corrective approaches.

## β‐Adrenergic receptor blockers

β‐Adrenergic receptor blockers (β‐blockers) have been described extensively in the literature as a treatment of symptomatic HCM since the 1960s. β‐Blockers are capable of reducing LVOT obstruction, angina, dyspnoea and the risk of ventricular arrhythmias (Spoladore *et al*. 2012). β‐Blockers inhibit sympathetic stimulation by binding to β‐AR (β‐adrenergic receptors) (Fig. 1). Downstream effects include decreased heart rate, contractility and LVOT obstruction (Spoladore *et al*. 2013). Studies performed in human induced pluripotent stem cell‐derived cardiac myocytes (hiPSC‐CMs) demonstrate some therapeutic effects of β‐blockers on myocyte hypertrophy, arrhythmia and Ca^2+^ handling abnormalities (Lan *et al*. 2013; Han *et al*. 2014; Toepfer *et al*. 2019*a*). Clinical studies in HCM patients indicate that β‐blockers reduce left ventricular diastolic pressures and improve left ventricular filling; however there appears to be little beneficial impact regarding long‐term effects on disease progression (Marian, 2009; Spoladore *et al*. 2012). Additionally, as β‐blockers are a broad class of therapeutics and are used for a variety of heart conditions, they carry the potential for adverse side effects (Farzam & Jan, 2020). Recent reviews on their efficacy indicate that chronic use of β‐blockers may induce additional side effects such as bradycardia, hypotension and atrioventricular nodal conduction block (Farzam & Jan, 2020).

## Metabolic modulating agents

While the healthy adult heart utilizes fatty acid oxidation as a primary source of energy production, hypertrophic and failing hearts shift toward glucose and lactate metabolism (Lopaschuk *et al*. 2010; Vakrou & Abraham, 2014). Additionally, reduced PCr/ATP ratios have been reported in HCM patients with established left ventricular hypertrophy and in patients before the development of the pathology (Jung *et al*. 1998; Crilley *et al*. 2003; Timmer *et al*. 2011; Abraham *et al*. 2013). These findings support the notion that excessive ATP utilization and subsequent energy deficiency is an early mechanism in the development of HCM pathology. With this, a number of studies have investigated the use of metabolic therapies to target energetic deficits (Lee *et al*. 2005; Abozguia *et al*. 2010; Horowitz & Chirkov, 2010; van Driel *et al*. 2019).

Over the past decade, metabolic modulating agents such as perhexiline, trimetazidine and ranolazine, which were initially developed as therapeutic agents for angina, have been examined as potential HCM therapeutics (Abozguia *et al*. 2010; Olivotto *et al*. 2018). Perhexiline is thought to bind to and inhibit mitochondrial carnitine palmitoyltransferase enzymes, shifting myocardial substrate utilization from fatty acid oxidation to glucose metabolism (Ashrafian *et al*. 2007). In a Phase 2 clinical trial (METAL‐HCM trial), perhexiline treatment (100 mg administered for 3–6 months) appeared to improve myocardial ratios of PCr/ATP ratio (indicative of improved energetics), diastolic dysfunction and $P_{vO_{2}}$ during exercise in a cohort of symptomatic HCM patients (Abozguia *et al*. 2010). Trimetazidine, a metabolic modulator and anti‐ischaemic agent, is believed to act via inhibition of fatty acid β‐oxidation, shifting metabolism from fatty acid oxidation to glucose oxidation (Dezsi, 2016; Steggall *et al*. 2017). In a Phase 2b clinical trial, trimetazidine (20 mg administered 3 times a day for 3 months) was shown to be ineffective in improving exercise capacity in symptomatic patients with non‐obstructive HCM (Coats *et al*. 2019). Ranolazine acts to inhibit fatty acid β‐oxidation and late inward sodium channels (Ardehali *et al*. 2012; Steggall *et al*. 2017). In a Phase 4 clinical trial, ranolazine (500 mg administered for 60 days) was used in the treatment of non‐obstructive HCM, and although it effectively relieved some symptomatic features (angina and dyspnoea), it was demonstrated to have no overall effect on exercise performance or diastolic dysfunction (Gentry *et al*. 2016; Olivotto *et al*. 2018). Although some improvements have been observed, there is conflicting evidence in relation to improvements in overall functional capacity of HCM patients, with a small number of studies reporting adverse side effects (Abozguia *et al*. 2010; Gentry *et al*. 2016; Olivotto *et al*. 2018). Overall, it would appear that enhancing myocardial glucose metabolism may not be an efficacious approach in the treatment of HCM.

## Calcium channel inhibitors

Calcium channel inhibitors target the pore‐forming α_1C_ subunit of the I_Ca‐L_ and have been used as an alternative treatment to β‐blockers in clinical settings (Fig. 1) (Striessnig *et al*. 2015). Calcium channel blockers are used in a similar manner to β‐blockers in that they reduce heart rate and contractility, leading to improved diastolic filling and outflow; however, they are primarily administered in patients that exhibit non‐obstructive HCM, or as an alternative in those experiencing adverse side effects with β‐blockers (Spoladore *et al*. 2013; Striessnig *et al*. 2015). Calcium channel blockers such as diltiazem interrupt Ca^2+^ dysregulation processes through attenuation of Ca^2+^‐induced Ca^2+^ release, and subsequent restriction of Ca^2+^ uptake by the mutated sarcomere (Semsarian *et al*. 2002). Calcium channel inhibitors also cause greater negative ionotropic effects compared to β‐blockers due to the inhibition of Ca^2+^ through the channel pore and thereby tend to lead to poor clinical outcomes (Braunwald *et al*. 2002; Ho *et al*. 2015).

### Animal studies

Studies performed in a mouse model of HCM due to a *Tnnt2* mutation have revealed that in this model diastolic dysfunction occurs in the absence of significant hypertrophy (Westermann *et al*. 2006). Hypertrophy develops later in the pathogenesis of the disease. Under resting conditions, 21‐ to 30‐week‐old pre‐hypertrophic mice with *Tnnt2* mutation cTnT‐Ile79Asn, demonstrate left ventricular diastolic dysfunction, hypercontractility, enhanced myofilament Ca^2+^ sensitivity and cardiac stiffness, in the absence of hypertrophy or cardiac interstitial fibrosis. In response to β‐adrenergic stimulation (isoproterenol), cTnT‐Ile79Asn mice exhibit diastolic HF and SCD (Westermann *et al*. 2006). However, when pre‐treated with diltiazem (25 mg kg^−1^ day^−1^), isoproterenol‐induced HF and SCD was prevented (Table 1). It was proposed that this effect may have been due to acute inhibition of I_Ca‐L_ current, resulting in reduced Ca^2+^ influx into myocytes, and subsequent alterations in diastolic Ca^2+^ (Westermann *et al*. 2006). Certainly, it would appear that pre‐treatment of the cTnT‐Ile79Asn mice with diltiazem prevented isoproterenol‐induced HF and SCD.

**Table 1:** Efficacy of current treatments in models of hypertrophic cardiomyopathy

Gene	Mutation/Model	*in vitro/in vivo*	Pre/Post HCM	Characteristics	Treatment	Outcomes	Ref
Animal models							
* TNNT2*	*Tnnt2*‐TnT‐I79N mice	*in vivo*	Pre (21–30 week) ISO	HF/SCD: ↑	Diltiazem (25 mg kg^−1^ day^−1^, 50 days)	HF/SCD: ↓	Westermann* et al*. (2006)
* MYBPC3*	Homozygous *Mybpc3* KI (c.772G>A) mice	*in vitro*	Post (32–34 week) ISO/Paced	Sarcomere length: ↓ Ca^2+^ transient time to peak: ↑ Arrhythmias: ↑	Diltiazem (1 µm)	Sarcomere length: ↑ Ca^2+^ transient time to peak: ↓ Arrhythmias: ↓	Flenner *et al*. (2017)
		*in vivo*	Post (6–8 week)	Hypertrophy & Dysfunction: ↑ Fibrosis: ↑	Diltiazem (25 mg kg^−1^ day^−1^, 6 months)	Hypertrophy and Dysfunction: ↑ (no Δ) Fibrosis: ↑ (no Δ)	
	*Mybpc^t/+^ Mybpc^t/t^* mice	*in vitro*	Post (8–20 week)	*MYBPC3^t/+^* Cell shortening: ↑ *MYBPC3^t/t^* Cell shortening: ↑ Relaxation time: ↑	MYK‐461 (0.15 µm, 0.3 µm)	*MYBPC3^t/+^* Cell shortening: ↓ LVWT: not reported *MYBPC3^t/t^* Cell shortening: ↓ Relaxation time: ↓ LVWT: not reported	Toepfer *et al*. (2019*b*)
* TNNI3*	*TnnI3‐*Gly203Ser mice	*in vitro*	Pre (10–15 week)	MMA: ↑ Ψ_m_: ↑	Nisoldipine (15 µm)	MMA: ↓ Ψ_m_: ↓	Viola *et al*. (2016*a*)
			Post (25–30 week)	Ψ_m_: ↑	Diltiazem (15 µm)	Ψ_m_: ↓	
				MMA: ↑ Ψ_m_: ↑	Nisoldipine (15 µm)	MMA: ↓ Ψ_m_: ↓	
		*in vivo*	Pre (20 week)	I_Ca‐L_ inactivation rate: ↑ MMA: ↑ Ψ_m_: ↑	AID‐TAT (10 µm, 3×/week/5 week)	I_Ca‐L_ inactivation rate: ↓ MMA: ↓ Ψ_m_: ↓ Myocyte hypertrophy: ↓ HW:BW: ↓ IVST: ↓ LVEDD/LVESD: ↑ FS: ↓	Viola *et al*. (2020)
			Post (30 week)	I_Ca‐L_ inactivation rate: ↑ MMA: ↑ Ψ_m_: ↑ Myocyte hypertrophy: ↑ HW:BW: ↑ IVST: ↑ LVEDD/LVESD: ↓ FS: ↑	AID‐TAT (10 µm, 3×/week/5 week)	MMA: ↑ (no Δ) Ψ_m_: ↑ (no Δ) Myocyte hypertrophy: ↑ (no Δ) HW:BW: ↑ (no Δ) IVST: ↑ (no Δ) LVEDD/LVESD: ↓ (no Δ) FS: ↑ (no Δ)	
* MYH7*	*MYH7*‐Arg403Gln mice	*in vitro*	Pre (10–15 week)	MMA: ↑ Ψ_m_: ↑	Nisoldipine (15 µm)	MMA: ↓ Ψ_m_: ↓	Viola *et al*. (2016*b*)
			Post (30–50 week)	Ψ_m_: ↑	Diltiazem (15 µm)	Ψ_m_: ↓	
				MMA: ↑ Ψ_m_: ↑	Nisoldipine (15 µm)	MMA: ↓ Ψ_m_: ↓	
		*in vivo*	Pre (10–15 week)	Ca^2+^‐binding proteins: ↓ Fibrosis: ↑ Myocyte hypertrophy & disarray: ↑	Diltiazem (25 mg kg^−1^ day^−1^, 7 weeks)	Ca^2+^‐binding proteins: ↑ Fibrosis: ↓ Myocyte hypertrophy and disarray: ↓	Semsarian *et al*. (2002)
			Post (30–50 week)	Hypertrophic markers: ↑ Myocyte disarray: ↑ Fibrosis: ↑ ESV and EDV: ↓ FS: ↑		Hypertrophic markers: ↓ Myocyte disarray: ↓ Fibrosis: ↓ ESV and EDV: ↑ FS: ↑ (no Δ)	
	*MYH7 –* Arg403Gln Arg719Trp Arg453Cys mice	*in vivo*	Pre (6–15 week)	NA	MYK‐461 (2.5 mg kg^−1^ day^−1^, 20–26 weeks)	LVWT: improved (↓) FS: improved (↓) Fibrosis: improved (↓) Myocyte disarray: improved (↓)	Green *et al*. (2016)
			Post (30–35 week)	LVWT: ↑ FS: ↑ Fibrosis: ↑ Myocyte disarray: ↑	MYK‐461 (2.5 mg kg^−1^ day^−1^, 4 weeks)	LVWT: partial ↓ FS: partial ↓ Fibrosis: ↑ (no Δ) Myocyte disarray: ↑ (no Δ)	
Not specified	Idiopathic HCM felines	*in vivo*	Post (5.7–10.8 years)	LVWT: ↑ IVST: ↑ LVEDD: ↓ FS: ↑	Diltiazem (∼5.34 mg kg^−1^, 6 months)	LVWT: ↓ IVST: ↓ LVEDD: ↑ FS: ↑ (no Δ)	Bright *et al*. (1991)
					Verapamil (∼5.25 mg kg^−1^, 6 months)	Adverse effects or HF/SCD	
					Propanolol (∼2 mg kg^−1^ day^−1^, 6 months)	Adverse effects or HF/SCD	
Not specified	Idiopathic HOCM felines	*in vivo*	Post (0.9–3.7 years) ISO	FS: ↑ LVOT obstruction: ↑ SAM: ↑	MYK‐461 (0.12‐0.36 mg kg^−1^ h^−1^)	FS: ↓ LVOT obstruction: ↓ SAM: ↓ LVWT: not reported	Stern *et al*. (2016)
hiPSC‐CM studies							
* MYBPC3*	*Mybpc^t/+^*	*in vitro*	30 days post‐differentiation	Cell shortening: ↑ Contractility: ↑ Relaxation time: ↑	Propanolol (0–10 µm l^−1^)	Cell shortening: ↓ Contractility and relaxation time: ↑ (no Δ)	Toepfer *et al*. (2019*a*)
					Verapamil (0–10 µm l^−1^)	Cell shortening: ↓ Contractility and relaxation time: ↑ (no Δ, except with high concentrations, ↓)	
					MYK‐461 (0–10 µm l^−1^)	Cell shortening: ↓ Contractility: ↓ Relaxation time: ↑ (no Δ, except with high concentrations, ↓)	
* MYH7*	*MYH7 –* Arg663His	*in vitro*	20–40 days post‐differentiation ISO	Hypertrophic markers: ↑ Myocyte hypertrophy: ↑ Ca^2+^ handling abnormalities: ↑ Arrhythmia: ↑	Propanolol (400 nm)	Myocyte hypertrophy: ↓ Ca^2+^ handling abnormalities: ↓ Arrhythmia: ↓	Lan *et al*. (2013)
					Verapamil, (50–100 nm)	Myocyte hypertrophy: ↓ Ca^2+^ handling abnormalities: ↓ Arrhythmia: ↓	
					Diltiazem, (50–100 nm)	Ca^2+^ handling abnormalities: ↓ Arrhythmia: ↓	
	*MYH7 –* Arg442Gly	*in vitro*	30 days post‐differentiation ISO	Contractility: ↑ Myocyte hypertrophy & disarray: ↑ Ca^2+^ handling abnormalities: ↑ Arrhythmia: ↑	Metapropolol (10 µm)	Arrhythmia: ↓	Han *et al*. (2014)
					Verapamil (100 nm)	Ca^2+^ handling abnormalities: ↓ Arrhythmia: ↓	
Human studies							
* MYBPC3*	*MYBPC3 –* Q969X, N755K human patients	*in vivo*	Pre (20–55 years)	*S*′: ↓ *E*′: ↓	Diltiazem (240 mg day^−1^, 8 weeks)	*S*′: ↑ *E*′: ↑	McTaggart (2004)
* MYH7* * MYBPC3* * TNNT2*	Mixture of 25 mutations	*in vivo*	Pre (5–39 years)	NA	Diltiazem (5 mg kg^−1^ day^−1^, 12–42 months)	LVWT dimension: improved (↓) LVEDD: improved (↑) *MYBPC3* LVWT: improved (↓) *E*/*E*′: improved (↓) cTnI: improved (↓)	Ho *et al*. (2015)
Not specified	Human HOCM patients	*in vivo*	Post (22–70 years) PIONEER HCM TRIAL (Phase 2)	Resting LVEF: ↑ Post‐exercise LVOT gradient: ↑ LVWT: ↑	MYK‐461 (10–20 mg day^−1^, 12 weeks)	Resting LVEF: ↓ Post‐exercise LVOT gradient: ↓	
LVWT: not reported							Heitner *et al*. (2019)
Not specified	Human HCM patients	*in vivo*	Post (mean age: 54 years) MAVERICK HCM trial (Phase 2)	LVEF: ↑ NT‐proBNP (wall stress): ↑	19, 21, 19 patients to 200 ng ml^−1^, 500 ng ml^−1^, or placebo, respectively	LVEF: ↓ (in 5 *MYK‐461* patients) NT‐proBNP (wall stress): ↓ cTnI: ↓ LVWT: not reported	Ho *et al*. (2020*a*)
Unknown	Human HOCM patients	*in vivo*	>18 years EXPLORER HCM trial (Phase 3)	LVEF: ↑ $P_{vO_{2}}$: ↑ LVOT gradient: ↑	2.5, 5.0, 10.0 or 15 mg day^−1^, 30 weeks	LVEF: TBA $P_{vO_{2}}$: improved (↓) Post‐exercise LVOT gradient: improved (↓) LVWT: not reported	Ho *et al*. (2020*b*), Myokardia (2020)

*E*′, peak velocity of early diastolic mitral annular motion; *E*/*E*′, ratio of peak velocity of early diastolic transmitral flow to mitral annular motion; EDV, end‐diastolic volume; ESV, end‐systolic volume; FS, fractional shortening; HW:BW, heart weight to body weight ratio; HOCM, obstructive hypertrophic cardiomyopathy; HF, heart failure; I_Ca‐L_, L‐type calcium channel; ISO, isoproterenol; IVST, Intraventricular septum thickness; LVD, left ventricular diameter; LVEDD, left ventricular end‐diastolic diameter; LVEDP, left ventricular end‐diastolic pressure; LVEF, left ventricular ejection fraction; LVESD, left ventricular end‐systolic diameter; LVOT, left ventricular outflow tract obstruction; LVWT: left ventricular wall thickness; MMA, mitochondrial metabolic activity; NA, not applicable; Ψ_m_, mitochondrial membrane potential; $P_{vO_{2}}$, peak oxygen consumption; *S*′, systolic velocity peak; SAM, systolic anterior motion of the mitral valve; SCD, sudden cardiac death; Δ, change.

Although the I_Ca‐L_ is the primary target of diltiazem, it is also known to have other cellular targets including the mitochondrial Na^+^/Ca^2+^ exchanger (Striessnig *et al*. 2015). With this, it has been proposed that diltiazem may reduce hypertrophic presentation by normalizing alterations in mitochondrial Ca^2+^ concentration, thereby improving cardiac energetics (Semsarian *et al*. 2002). In a recent study, diltiazem was assessed as an HCM therapeutic in homozygous *Mybpc3*‐targeted knock‐in (KI) mice carrying a c.772G>A transition on the last nucleotide of exon 6 (*Mybpc3* KI (c.772G>A)) (Fraysse *et al*. 2012; Flenner *et al*. 2017). *Mybpc3* KI (c.772G>A) mice exhibit increased systolic and diastolic dysfunction and myofilament Ca^2+^ sensitivity followed by cardiac hypertrophy (Fraysse *et al*. 2012; Flenner *et al*. 2017). Cardiac myocytes were isolated from cardiomyopathic *Mybpc3* KI (c.772G>A) mice, and exposed to isoproterenol and high pacing frequency stress conditions (Flenner *et al*. 2017). Under these conditions, myocytes exhibited decreased diastolic sarcomere length, increased Ca^2+^ transient rise, and arrhythmias (Flenner *et al*. 2017). Each of these observations was normalized in the presence of diltiazem (Table 1). *In vivo* studies were also performed in 6‐ to 8‐week‐old pre‐cardiomyopathic *Mybpc3* KI (c.772G>A) mice, treated with diltiazem for 6 months (Flenner *et al*. 2017). Diltiazem treatment did not prevent activation of the fetal gene programme, cardiac hypertrophy and dysfunction, or fibrosis (Table 1) (Flenner *et al*. 2017). These data suggest that while acute diltiazem treatment in post‐hypertrophic *Mybpc3* KI (c.772G>A) mice may be beneficial in prevention of stress‐induced contractile abnormalities, chronic administration of diltiazem treatment does not appear to reverse or prevent development of HCM pathology.

Studies performed in αMHC^403/+^ mice have indicated abnormal Ca^2+^ handling and reduced Ca^2+^‐binding and storage protein levels in this model, including calsequestrin, junctin, triadin and ryanodine receptor 2 (RyR2) compared to control mice (Semsarian *et al*. 2002). This occurs before the onset of the disease phenotype. In the same study, 15‐ to 20‐week‐old pre‐hypertrophic αMHC^403/+^ mice were treated with diltiazem (25 mg kg^−1^ day^−1^). Following 7 weeks of treatment, Ca^2+^‐binding and storage protein levels were restored (Table 1) (Semsarian *et al*. 2002). Interestingly, histological features such as fibrosis, myocyte hypertrophy and disarray were also abated with early diltiazem treatment (Semsarian *et al*. 2002). Similar studies were performed in 30‐ to 50‐week‐old post‐hypertrophic αMHC^403/+^ mice. Following 7 weeks of diltiazem treatment, αMHC^403/+^ mice demonstrated reduced expression of hypertrophic molecular markers, reduced left ventricular wall thickness, improved end‐diastolic and end‐systolic volumes, and reduced fibrosis, compared to untreated mice (Table 1) (Semsarian *et al*. 2002). However, fractional shortening (FS) was not improved. These data indicate that an early, pre‐treatment approach may be more effective in preventing HCM pathology.

*In vitro* studies performed in cardiac myocytes isolated from both pre‐ and post‐cardiomyopathic αMHC^403/+^ mice provide additional support for an early intervention approach. Myocytes isolated from both pre‐ and post‐cardiomyopathic αMHC^403/+^ mice exhibit a significantly faster I_Ca‐L_ inactivation rate, and subsequently a hypermetabolic mitochondrial state in response to BayK(−), compared to myocytes isolated from age‐matched wild‐type mice (Viola *et al*. 2016*b*). Exposure of αMHC^403/+^ myocytes to diltiazem or nisoldipine (15 µm) normalized mitochondrial metabolic activity in both pre‐ and post‐cardiomyopathy αMHC^403/+^ myocytes (Table 1). Similar findings have been observed in the cTnI‐G203S mouse model (Viola *et al*. 2016*a*). It has been proposed that early diltiazem treatment may restore structural–functional communication between the I_Ca‐L_ and mitochondria, and that subsequent restoration of mitochondrial metabolic activity may prevent the development of HCM disease progression.

A comparative study performed in an idiopathic feline model of HCM examined the efficacy of diltiazem and verapamil compared to β‐blockers such as propranolol (Bright *et al*. 1991). In this model, diltiazem, verapamil or propranolol were administered to post‐hypertrophic felines that exhibited significant left ventricular hypertrophy and impaired diastolic function, for up to 6 months (Bright *et al*. 1991). All 12 cats receiving chronic diltiazem treatment demonstrated alleviation of clinical symptoms, including left ventricular wall thickness (LVWT), intraventricular septum thickness (IVST) and left ventricular end‐diastolic diameter (LVEDD) (Table 1) (Bright *et al*. 1991). However, FS was not improved. The survival rate for the 6‐month study duration was 94% in the diltiazem treatment group, 50% for Verapamil and 33% for propranolol (Bright *et al*. 1991). Cats receiving propanalol and verapamil treatment experienced severe adverse side effects or they died due to HF/SCD, and therefore clinical data for these groups were not able to be reported (Bright *et al*. 1991). This would suggest that corrective diltiazem treatment may be a safe approach to relieve some HCM‐associated characteristics.

### hiPSC‐CM studies

*In vitro* experiments utilizing hiPCS‐CMs expressing a missense mutation in β‐MHC demonstrate numerous disease features of HCM, including cellular enlargement, contractile arrhythmia, Ca^2+^ dysregulation and sarcomeric disorganization (Lan *et al*. 2013; Han *et al*. 2014; Tanaka *et al*. 2014). In a hiPSC‐CM model expressing *MYH7* gene mutation Arg663His, *in vitro* verapamil prevented myocyte hypertrophy and abolished Ca^2+^ dysregulation and arrhythmias, while diltiazem exposure ameliorated Ca^2+^ handling abnormalities and arrhythmias, in single and multi‐cell preparations (Lan *et al*. 2013) (Table 1). Similar findings were observed in hiPSC‐CMs expressing *MYH7* gene mutation Arg442Gly, whereby *in vitro* exposure to verapamil normalized Ca^2+^ handling abnormalities and arrhythmias (Han *et al*. 2014).

### Human studies

Transgenic rabbit models of HCM and human clinical studies indicate that early diastolic velocities are abnormally low in *MYBPC3* gene mutation carriers before the development of left ventricular hypertrophy (Nagueh *et al*. 2001; Ho *et al*. 2002). Therefore, utilizing tissue Doppler, the effects of diltiazem treatment have been assessed in patients carrying *MYBPC3* gene mutations (Gln969X or Asn755Lys) (McTaggart, 2004). Patients were pre‐hypertrophic, with no symptomatic manifestations of HCM assessed by echocardiography and electrocardiography. Patients administered diltiazem (240 mg day^−1^) for 8 weeks, exhibited increases in both systolic velocity peak (*S′*) and early diastolic velocity peak (*E′*) that appeared to normalize cardiac flow, as compared to patients receiving placebo treatment (Table 1) (McTaggart, 2004). These data indicate a potential benefit of early diltiazem treatment in the pre‐hypertrophic stages of the disease in patients carrying *MYBPC3* gene mutations. Interestingly, the greatest improvements were in the youngest patients who may have had fewer structural changes present.

More recently, a pilot study was undertaken to assess the efficacy of diltiazem in preventing the phenotypic presentation of HCM in 38 patients carrying *MYBPC3*, *MYH7* and *TNNT2* gene mutations (Ho *et al*. 2015). Mutation carriers with no clinical diagnosis of HCM (specifically left ventricular hypertrophy as assessed by echocardiography) received chronic diltiazem treatment (5 mg kg^−1^ day^−1^, 12–42 months) or an equivalent placebo. Patients treated with diltiazem exhibited improved LVWT‐to‐dimension ratio, and LVEDD, compared to the placebo group (Table 1) (Ho *et al*. 2015). Within the diltiazem‐treated *MYBPC3* mutation carriers, LVWT, diastolic filling (reflected by *E*/*E′*) and cardiac troponin I levels were improved compared to the placebo group (Ho *et al*. 2015). Interestingly, four unrelated patients, three with *MYH7* mutations and one with a *TNNT2*, did not respond to diltiazem treatment.

Overall, studies performed in animal models of HCM and human clinical studies indicate that early treatment with diltiazem may be beneficial to prevent some HCM‐associated characteristics. Mechanistically, this may occur by normalizing cellular Ca^2+^ handling, and/or by restoring structural–functional communication between the I_Ca‐L_ and mitochondria and subsequently normalizing mitochondrial metabolic activity. Certainly, treatment efficacy appears to vary depending on the underlying gene mutation.

## MYK‐461

Patients with HCM often present with early hypercontractility that stems from a high degree of actin–myosin cross‐linking (Heitner *et al*. 2019). Recent studies have identified a cardiac‐specific small‐molecule, mavacamten (MYK‐461), that directly targets the sarcomere by modulating β‐MHC (Green *et al*. 2016; Stern *et al*. 2016; Kawas *et al*. 2017). This reversibly inhibits β‐MHC–actin binding, and subsequently reduces sarcomere force output and contractility (Fig. 1) (Heitner *et al*. 2019). Over the past 5 years, several studies have investigated the efficacy of MYK‐461 as a potential HCM therapeutic.

### Animal models

The effectiveness of MYK‐461 treatment has been assessed in murine models of HCM expressing cMyBP‐C gene mutations (*Mybpc3^t/+^* and *Mybpc3^t/t^*) (Toepfer *et al*. 2019*b*). Echocardiography studies have revealed that Mybpc3^t/+^ (endogenous heterogyzous) mice exhibit minimal increases in left ventricular posterior wall thickness, and depressed cardiac contractility compared to wild‐type mice (Toepfer *et al*. 2019*b*) (Table 1). On the other hand, Mybpc3^t/t^ (homozygous truncated) mice exhibit significantly increased left ventricular volumes and mass, but depressed contractile function (Toepfer *et al*. 2019*b*). However, studies performed in isolated cardiac myocytes revealed contractile differences that were not apparent from *in vivo* echocardiography. Utilizing isolated cardiac myocytes, sarcomere length was measured throughout the contractile cycle to assess *in vitro* contractility and relaxation (defined as proxies for systolic and diastolic function respectively). Cardiac myocytes isolated from Mybpc3^t/+^ and Mybpc3^t/t^ mice exhibited significantly increased cell shortening compared to wild‐type myocytes (Toepfer *et al*. 2019*b*). Relaxation time was significantly increased in Mybpc3^t/t^ myocytes, but not significantly altered in Mybpc3^t/−^ myocytes. These data are consistent with a hypercontractile state. Acute exposure to MYK‐461 (0.15–0.3 µm) significantly reduced cell shortening in both Mybpc3^t/+^ and Mybpc3^t/t^, and normalized relaxation times in Mybpc3^t/t^ myocytes (Toepfer *et al*. 2019*b*). These *in vitro* data indicate that MYK‐461 may normalize contractile function.

Studies have also been performed in mice expressing β‐MHC mutations to investigate the efficacy of MYK‐461 in both preventing and reversing associated HCM (Green *et al*. 2016). Treatment of 6‐ to 15‐week‐old pre‐hypertrophic mice expressing β‐MHC mutations (Arg403Gln, Arg719Trp or Arg453Cys) with MYK‐461 (2.5 mg kg^−1^ day^−1^, for 20–26 weeks) reduced LVWT and FS, reduced fibrosis and improved myocyte organization compared to untreated mutant counterparts (Green *et al*. 2016) (Table 1). Treatment of 30‐ to 35‐week‐old cardiomyopathic mice with MYK‐461 (2.5 mg kg^−1^ day^−1^ for 4 weeks) was associated with a significant reduction in FS and LVWT by 2 and 4 weeks, respectively; however, these parameters remained stable with no further improvements observed for the remainder of the study (Green *et al*. 2016). Additionally, no significant reduction in fibrosis or myocyte disarray was observed.

Idiopathic feline models of obstructive HCM (HOCM) exhibit LVH, myocyte disarray, fibrosis, HF and SCD (Stern *et al*. 2016). At rest, felines predisposed to HOCM exhibit systolic anterior motion and LVOT obstruction (Stern *et al*. 2016). A recent study used a post‐cardiomyopathic model to examine the efficacy of MYK‐461 in isoproterenol stress responses (Stern *et al*. 2016). Felines administered MYK‐461 (0.12–0.36 mg kg^−1^ h^−1^) before isoproterenol treatment exhibited a significantly reduced FS compared to vehicle treatment, without negatively impacting heart rate (Stern *et al*. 2016) (Table 1). In addition, in post‐hypertrophic felines exposed to isoproterenol, MYK‐461 treatment reduced systolic anterior motion of mitral valves and prevented worsening of LVOT obstruction (Stern *et al*. 2016). Overall, early MYK‐461 treatment appeared to improve contractility and relieve inducible HOCM (Stern *et al*. 2016).

### hiPSC‐CM studies

An *in vitro* model utilizing hiPCS‐CMs that express a heterozygous truncation variant in the *MYBPC3* gene (*Mybpc^t/+^*) recapitulates aspects of the HCM phenotype including hypercontractility, cell shortening and impaired relaxation (Toepfer *et al*. 2019*a*). Consistent with observations in animal models of the disease, exposure of hiPSC‐CMs to MYK461 resolved contractile abnormalities, specifically low doses (1 µmol l^−1^) normalized hypercontractility, whereas higher doses (2–4 µmol l^−1^) were required to normalize relaxation times (Toepfer *et al*. 2019*a*) (Table 1).

### Human studies

In a Phase 2 clinical trial (PIONEER HCM trial), patients with symptomatic HOCM presenting with elevated resting left ventricular ejection fraction (LVEF), post‐exercise left ventricular outflow tract (LVOT) obstruction and LVWT, received MYK‐461 (10–20 mg day^−1^) for 12 weeks (Heitner *et al*. 2019). Patients receiving MYK‐461 treatment demonstrated improved resting LVEF (reduced), improved peak oxygen consumption (increased), and reduced post‐exercise LVOT gradients (Heitner *et al*. 2019) (Table 1). The effect of MYK‐461 on LVWT was not reported.

In another Phase 2 clinical trial (MAVERICK‐HCM trial), patients with symptomatic non‐obstructive HCM (characterized by the presence of hyper‐contractility and impaired relaxation but no significant LVOT obstruction at rest or with provocation) were treated with 200 ng ml^−1^, 500 ng ml^−1^ or a placebo for 16 weeks (Ho *et al*. 2020*a*). Compared to placebo‐treated individuals, patients receiving MYK‐461 treatment exhibited improved LVEF (reduced), improved peak oxygen consumption ($P_{vO_{2}}$) and decreased wall stress as indicated by reduced levels of serum biomarkers such as N‐terminal pro‐B‐type natriuretic peptide (NT‐proBNP) (Ho *et al*. 2020*a*) (Table 1). No datve has been released regarding the effect of MYK‐461 on LVWT.

A recent Phase 3 trial (EXPLORER HCM trial) was undertaken involving patients with HOCM presenting with LVOT obstruction and associated left ventricular hypertrophy, being administered MYK‐461 at a range of doses (2.5, 5, 10 and 15 mg day^−1^) over a 30‐week period (Ho *et al*. 2020*b*). Primary and secondary efficacy assessments included post‐exercise LVOT peak gradient, $P_{vO_{2}}$ and serum biomarkers of myocardial wall stress (NT‐proBNP, cTnI). Sub‐study evaluations included LVWT, myocardial fibrosis and cardiac chamber volume/function (Ho *et al*. 2020*b*) (Table 1). To date, patients receiving MYK‐461 have been reported to display improvements in $P_{vO_{2}}$ and LVOT gradient (decreased) (Myokardia, 2020). No data have been released regarding the effect on LVWT (Myokardia, 2020).

The use of hiPSC‐CMs has been an important development in the field of cardiovascular disease modelling to further understand pathophysiological mechanisms of cardiovascular diseases *in vitro*, and develop novel therapeutic treatments for cardiovascular diseases such as HCM (Lodrini *et al*. 2020). The recent application of genome editing to hiPSC‐CMs has enabled further investigation on the genetic causation of HCM. However, limitations exist, as hiPSC‐CMs are structurally and functionally immature in comparison to human adult cardiac myocytes and therefore do not fully recapitulate their complex physiological properties (Lan *et al*. 2013; Han *et al*. 2014; Ramachandra *et al*. 2019). Nonetheless, when considered together, studies performed in animal models of HCM, hiPSC‐CM and human clinical studies indicate that early therapeutic intervention with MYK‐461 may be effective in normalizing HCM‐associated hypercontractility, and relieve inducible HOCM, by inhibiting β‐MHC–actin binding and subsequently, reducing sarcomere force output.

## AID‐TAT peptide

Studies utilizing transgenic mouse models of HCM indicate that a structural–functional ‘breakdown’ between the cardiac I_Ca‐L_ and mitochondria via sarcomeric proteins may lead to the development of a hypermetabolic mitochondrial state, which precedes development of HCM pathology (Viola *et al*. 2016*a*,*b*). Recent studies have investigated the use of AID‐TAT peptide as a potential HCM therapeutic (Viola *et al*. 2020). Unlike β‐blockers and Ca^2+^ channel blockers, AID‐TAT peptide specifically targets the AID region of the cardiac I_Ca‐L_, immobilizing movement of the I_Ca‐L_ β_2_ subunit (Fig. 1) (Hohaus *et al*. 2000; Viola *et al*. 2020). Twenty‐week‐old pre‐cardiomyopathic cTnI‐G203S mice were treated with AID‐TAT peptide (10 µm) three times a week for 5 weeks (Viola *et al*. 2020). Treatment with AID‐TAT peptide resulted in significant improvements in cellular I_Ca‐L_ kinetics, mitochondrial metabolic activity and cell size (decreased), and a significant decrease in heart weight to body weight ratio (Viola *et al*. 2020). *In vivo* echocardiography revealed a significant improvement in LVEDD/LVESD (increase), and IVST and FS (decrease) in cTnI‐G203S mice treated with AID‐TAT peptide. Treatment of 30‐week‐old post‐cardiomyopathic cTnI‐G203S mice with established hypertrophy with AID‐TAT peptide did not significantly improve mitochondrial metabolic activity, cell size, heart weight to body weight ratio (HW:BW) or echocardiographic parameters (Viola *et al*. 2020). These studies indicate that early therapeutic intervention with AID‐TAT peptide may represent a viable approach to restore structural–functional communication between I_Ca‐L_ and mitochondria, normalize metabolic activity and prevent the development of HCM.

## Conclusion

Conventionally, HCM is characterized by cardiac myocyte remodelling, disorganization of sarcomeric proteins, interstitial fibrosis and altered energy metabolism. There is now evidence to suggest that alterations in Ca^2+^ handling, energy metabolism, contractility and sarcomeric disorganization may precede the presentation of hypertrophy and fibrosis. Indeed, here we find that a preventative rather than corrective therapeutic approach may be more efficacious in the treatment of HCM. However, while some similarities exist, each mutation appears to lead to mutation‐specific pathophysiology, which may contribute to the observed clinical phenotypic variability in sarcomere‐related HCM (Viola & Hool, 2019). A clear understanding of early mutation‐specific mechanisms may be required, on a cellular level, in order to determine the most effective therapeutic mode of action. Studies investigating the efficacy of diltiazem or AID‐TAT peptide indicate that early treatment may be beneficial in preventing hypertrophy by normalizing cellular Ca^2+^ handling, and/or normalizing mitochondrial metabolic activity. On the other hand, early therapeutic intervention with MYK‐461 may be effective in normalizing hypercontractility and relieve inducible HOCM, by reducing sarcomere force output. In addition to mutation‐specific pathophysiology, epigenetic differences, genetic modifiers and environmental factors can also influence HCM morphology, producing a variety of clinical phenotypes from the same gene mutation (Burke *et al*. 2016). Therefore, an understanding of the physiological mechanisms underlying patient‐specific pathology will also be an important consideration in the design of personalized treatment approaches, or ‘precision medicine’ (Dainis & Ashley, 2018), for HCM patients.

## Additional information

### Competing interests

None.

### Author contributions

All authors have read and approved the final version of this manuscript and agree to be accountable for all aspects of the work in ensuring that questions related to the accuracy or integrity of any part of the work are appropriately investigated and resolved. All persons designated as authors qualify for authorship, and all those who qualify for authorship are listed.

### Funding

This study was supported by the National Health and Medical Research Council of Australia [APP1143203, APP1103782]. Livia Hool is a National Health and Medical Research Council Senior Research Fellow [APP1117366]. Helena Viola is a Heart Foundation Future Leader Fellow [101930].
